# A Randomized Cohort Study: Is It Worth the Time to Receive Antiandrogenic Pretreatment Before Ovulation Induction for Women With Polycystic Ovary Syndrome?

**DOI:** 10.3389/fendo.2022.813188

**Published:** 2022-02-24

**Authors:** Zhiyan Chen, Jichun Tan, Huichun Wang, Beihong Zheng, Jian Liu, Guimin Hao, Zaixin Guo, Zhengyi Sun, Qi Yu

**Affiliations:** ^1^ Department of Obstetrics and Gynecology, National Clinical Research Center for Obstetric & Gynecologic Diseases, Peking Union Medical College Hospital, Chinese Academy of Medical Sciences & Peking Union Medical College, Beijing, China; ^2^ Department of Obstetrics and Gynecology, Shengjing Hospital of China Medical University, Shenyang, China; ^3^ Reproductive Medical Center, Haidian Maternal and Child Health Hospital, Beijing, China; ^4^ Reproductive Medicine Center, Fujian Maternity and Child Health Hospital, Affiliated Hospital of Fujian Medical University, Fuzhou, China; ^5^ Department of Obstetrics and Gynecology, Shuangliu Maternal and Child Health Hospital, Chengdu, China; ^6^ Department of Reproductive Medicine, The Second Hospital of Hebei Medical University, Shijiazhuang, China

**Keywords:** combined oral contraceptives, hyperandrogenism, ovulation induction, polycystic ovary syndrome, pregnancy outcomes

## Abstract

**Objective:**

To assess the effect of antiandrogenic pretreatment using combined oral contraceptives (COCs) before ovulation induction in infertile patients with polycystic ovary syndrome (PCOS) with hyperandrogenism.

**Design:**

Prospective, randomized open-labeled cohort study

**Setting:**

Multicenter

**Patients:**

PCOS patients with hyperandrogenism and requiring infertility treatments

**Interventions:**

Randomization to direct ovulation induction of letrozole (letrozole group) or ethinylestradiol/cyproterone acetate (EE/CPA) for 3 months and subsequent letrozole-induced ovulation (EE/CPA+ letrozole group). The maximum number of ovulation induction cycle was three to four.

**Main Outcome Measures:**

Ovulation rate, conception rate, ongoing pregnancy rate, and live birth rate were the main outcomes of the study.

**Results:**

There were no significant differences in the cumulative ovulation, conception, ongoing pregnancy, and live birth rates between the letrozole and EE/CPA+ letrozole groups (cumulative ovulation: 206/254 [81.10%] *vs*. 169/205 [82.44%], risk ratio [RR]= 1.09 [0.68,1.76], P=0.713; conception: 44/90 [48.89%] *vs*. 42/76 [55.26%], RR= 1.29 [0.70,2.38], P=0.413; ongoing pregnancy: 33/90 [36.67%] *vs*. 33/76 [43.42%], RR=1.33 [0.71,2.47], P=0.376; and live birth: 32/90 [35.56%] *vs*. 31/76 [40.79%], RR=1.25 [0.67, 2.34], P=0.489).

**Conclusions:**

The results of this study showed that COC pretreatment was not superior to direct letrozole-induced ovulation therapy in improving ovulation and pregnancy results in women with PCOS. There is no benefit to perform antiandrogenic therapy before ovulation induction in patients with PCOS in clinical practice.

**Clinical Trial Registration:**

www.clinicaltrials.gov, identifier ChiCTR1900022839

## Introduction

Polycystic ovary syndrome (PCOS) is the most prevalent endocrine disorder affecting approximately 6–15% of women of reproductive age (1). Reproduction problems, including anovulatory infertility and increased adverse pregnancy events, affect 40% of patients with PCOS (2). Hence, one of the priorities in the treatment of PCOS is to improve the fertility of patients, and tremendous endeavor has been made by clinicians in this regard.

Hyperandrogenism is a prominent clinical hallmark of PCOS, which plays a detrimental role in female fecundity and may be a treatment target for conquering the reproduction problems (1). Hyperandrogenism exerts a deleterious impact on granulosa cell activity and follicular growth, leading to follicle dysmaturity and subsequent sterility (3, 4). Previous studies have also revealed that hyperandrogenism is associated with an increased risk of preterm delivery and preeclampsia in patients with PCOS (5). Consequently, antiandrogenic therapy may help enhance pregnancy outcomes in patients with PCOS with androgen excess in the long run.

Pretreatment approaches before ovulation induction has been investigated by previous studies to improve pregnancy outcomes for the patients with PCOS, including lifestyle modification (6) and insulin sensitizing agents (7, 8). Given the potential role of antiandrogenic therapy for infertile PCOS women, antiandrogenic pretreatment might also assist in enhancing fertility for the patients.

However, controversy exists among researchers whether antiandrogenic therapy with combined oral contraceptives (COCs), such as ethinylestradiol/cyproterone acetate (EE/CPA), should be performed before starting ovulation induction. One study suggested that COC pretreatment could increase implantation and pregnancy rates (9). In contrast, another study demonstrated that no improvement in pregnancy outcomes could be verified after the implementation of COC pretreatment (10). Additionally, the time needed to produce a benefit from antiandrogenic therapy may reduce the woman’s remaining reproductive window, depleting both patience and valuable fecundity, since female age continues to be the most important predictor of infertility treatment failure (11). Due to the lack of high-quality evidence regarding antiandrogenic pretreatment, well-designed clinical studies are needed to address this clinical dilemma.

Therefore, we designed this multicentered prospective clinical study to evaluate whether delayed infertility therapy for the antiandrogenic pretreatment using EE/CPA has advantages over immediate therapy in PCOS patients with hyperandrogenism. Additionally, the effect of antiandrogenic pretreatment on patients with different clinical characteristics was assessed. This study aimed to assist clinicians in recommending possible treatment strategies for infertile PCOS patients with hyperandrogenism.

## Materials and Methods

### Study Design and Ethics Approval

This was a multicentered prospective randomized controlled trial (RCT) study, conducted in six centers from the south, west, north, and central regions of China according to the Consolidated Standards of Reporting Trials (CONSORT) (12). This study was registered in the Chinese Registry of Clinical Trials at www.clinicaltrials.gov (registration number: ChiCTR1900022839) and was approved by the Institutional Review Board (IRB) of Peking Union Medical College Hospital (No: JS-1796). All procedures involving human subjects were conducted in accordance with the institutional ethical standards of the research center and the 1964 Declaration of Helsinki and its subsequent modifications or equivalent ethical standards. Written informed consent was obtained from all participants who were provided with a clear explanation of the trial by the assistant of each center.

### Data Management and Randomization

The Peking Union Medical College Hospital served as the coordination center for the trial, which was in charge of all data input, management, and analysis. The first author and corresponding author are responsible for the correctness and completeness of data. No commercial support was provided for this study.

We utilized an online third-party data management system to perform data collection and randomization. A computer-based random number generator was applied to perform block randomization in a 1:1 ratio, with stratification according to the participating centers. A random order was kept for 6–16 repeats. The research assistants conducted randomization of the patients after recruitment. Clinicians who recruited the patients were blinded of the participants’ assignments. However, the assignment results could not be blinded to the assistants and patients due to the differences in the treatment method and duration between the two treatment groups. The data analysts who performed the final statistical analysis were blinded to the clinical information of the patients.

### Study Population

Eligible participants were recruited between May 2019 and December 2020. All subjects were diagnosed with PCOS according to the diagnostic criteria proposed by the Rotterdam consensus as follows: (1) clinical or chemical hyperandrogenism, presented by hirsutism, acne, or androgenic alopecia (13); (2) oligomenorrhea, defined as menstrual cycle length > 35 days with < 8 menstrual cycles per year or amenorrhea, defined as no menstrual bleeding for 6 months or longer (14); and/or (3) polycystic ovaries, characterized as having more than 12 antral follicles with a diameter of 10 mm or single ovarian volume > 10 cm^3^; (4) exclusion of other causes of ovulation dysfunction and hyperandrogenism, including prolactin diseases, adrenal hyperplasia, and Cushing’s syndrome. Additional inclusion criteria were as follows: (1) age, 18–38 years old; (2) BMI, 18.5–28 kg/m2; (3) normal uterus and at least one patent fallopian tube, verified by hysterosalpingography, sonohysterography, or intrauterine pregnancy history in the past 3 years; (4) a male partner with a minimum sperm concentration of 14 million per milliliter, as defined by the World Health Organization (15); (5) a commitment by the couples to engage in regular intercourse with the intention of pregnancy throughout the study; and (6) no contraindications or allergic reactions of COCs and letrozole.

We excluded patients who were using any form of COCs, steroid hormones, antiandrogen therapies, or insulin sensitizers within 3 months; patients complicated with abnormal hepatic or renal functions; and patients with cardiovascular diseases, blood or immune diseases, malignancy, or mental disorders.

### Intervention

At baseline, participants were randomly assigned at a 1:1 ratio to the following two treatment groups: Group 1(letrozole group), immediate ovulation induction using letrozole; and Group 2 (EE/CPA+ letrozole group), antiandrogenic pretreatment using Diane-35 (EE/CPA) for 3 months and subsequent ovulation induction using letrozole. Letrozole was started on the third day of the menstrual cycle and continued for 5 days. A maximum of three or four cycles of ovulation induction was provided to the patients. In both treatment groups, the dose of letrozole was increased in subsequent menstrual cycles, up to a maximum daily dose of 7.5mg, if the patients showed nonresponse to the drug, defined as monophasic basal body temperature, or a midluteal progesterone level of less than 3 ng/mL, or consequent ultrasound scanning revealed no dominant follicle expulsion (performed by a proficient sonographer) (16). At least two to four times of intercourse per week were required for participating couples, which were also recorded by the patients accordingly (17).

### Clinical Measurements

#### Demographic and Anthropometric Data of Participants

We collected demographic data, including age, education, occupation, and medical history of each participant, and anthropometric measurements, including body mass index (BMI) and waist-to-hip ratio (WHR). BMI was calculated according to the formula: BMI = weight (kg)/height (m)^2^. The WHR was determined by calculating the ratio of the standing waist circumference to hip circumference. Normal weight and overweight were classified according to the criteria of the World Health Organization, as BMI of 18.5–23.9 kg/m2, 24–28 kg/m^2^, respectively (18, 19).

#### Clinical Assessments of PCOS

Menstruation and clinical hyperandrogenism of the patients were evaluated by a senior physician in each center. We utilized the modified Ferriman–Gallwey (m-FG) scores to evaluate the density of terminal hair at nine different body sites, including the upper lip, chin, chest, upper back, lower back, upper abdomen, lower abdomen, arm and thigh. And hirsutism was identified with scores ≥ 8 (20). The severity of acne was assessed by counting the number of lesions and their spread on the face, back, and chest, classified as mild, moderate, moderate to severe, and severe (21).

### Biochemical Measurements

Fasting blood tests for hepatic and renal functions, complete blood count, and blood coagulation functions were conducted as the safety parameters for recruitment at baseline. Plasma glucose, total cholesterol, low-density lipoprotein cholesterol (LDL-cholesterol), and serum insulin were measured after recruitment. The standard 75-g oral glucose tolerance test (OGTT) was also implemented for the patients (22). Insulin resistance (IR) was quantified by the homeostasis model assessment for IR (HOMA-IR) as follows: HOMA-IR = fasting serum insulin (U/L) × fasting plasma glucose (mmol/L)/22.5 (23). IR was defined as HOMA-IR ≥ 2.63 (24).

### Assessment of Endocrine Parameters

Biochemical tests were performed 3–5 days following spontaneous menstruation to determine the levels of serum triiodothyronine (T3), luteinizing hormone (LH), follicle-stimulating hormone (FSH), total testosterone, sex hormone binding globulin (SHBG), and sulfated dehydroepiandrosterone (DHEAS). Free androgen index (FAI) was calculated according to the formula: FAI = total testosterone (nmol/L)/serum SHBG (nmol/L) × 100 (25). The intra-assay variations of employed testing techniques were 1.6–6.3%, and inter-assay variations 5.8–9.6%.

### Outcomes

Cumulative ovulation, conception, ongoing clinical pregnancy, and live birth rates were considered as the primary outcomes of the study. Secondary outcomes included ovulation rate per treatment cycle, conception rate per treatment cycle, average cycles taken to ovulation, letrozole dosage for ovulation, average cycles taken to pregnancy, and letrozole dosage for pregnancy. Ovulation was defined as a biphasic basal body temperature, or a midluteal progesterone level of more than 3 ng/mL, or dominant follicle expulsion under consequent ultrasound scanning (26). Conception was determined as a positive result of human chorionic gonadotropin (hCG) with the values > 10 mIU/mL following ovulation induction (27). Ongoing clinical pregnancy was defined as a clinical pregnancy that continued for 12 weeks of gestation (28). The definition of live birth was an infant born alive after 28 weeks of gestation. Preterm delivery was defined as birth occurring prior to 37 weeks of gestation (29).

### Sample Size Calculation

We estimated that live birth rate of the immediate ovulation induction group (letrozole group) and the COC pretreatment group (EE/CPA+ letrozole group) was 20% and 40%, respectively. In this study, we aimed to detect the difference in live birth proportion between the two groups with 80% power and 5% two-sided significance. We calculated that a sample size of 81 per treatment group was needed using Pearson’s chi-square test. And we expanded to 115 per group to account for a 30% dropout rate.

The formulation for calculating sample size:


$$\text{n}=\frac{2\bar{\text{p}}\bar{\text{q}}({\left({\text{Z}}_{\alpha }+{\text{Z}}_{\beta }\right)}^{2}}{{\left(\text{p}1-\text{p}2\right)}^{2}}$$

n, sample size; p1, live birth rate of letrozole group; p2, live birth rate of EE/CPA+ letrozole group; $\bar{p}$ , mean of p1 and p2; $\bar{q}$ , mean of (1-p1) and (1-p2); With an α of 0.05 and a two-sided Z, Z_α_ = 1.96; with a β of 0.8, Z_β_ = 0.84.

### Statistical Analysis

Continuous variables were expressed as mean with standard deviation (SD) when normally distributed, or as median with interquartile range if not. Frequency and percentage values were calculated to express categorical variables. The Student’s t-test was used to compare continuous variables. The Chi-squared test was used to compare categorical variables.

A log-binomial regression model was used to compare the letrozole and EE/CPA+ letrozole groups for binary outcomes (e.g., ovulation, conception, ongoing pregnancy and live birth). Due to the prospective nature of our research, the log-binomial model allowed us to estimate the risk ratio (RR) rather than the odds ratio (OR). We categorized the patients into two groups according to their age(<30, ≥30), BMI(18.5–23.9, 24–28), and HOMA-IR(<2.63,≥2.63), and three groups based on the FAI tertile, to evaluate the effect of COC pretreatment on PCOS patients with different clinical characteristics.

We utilized Kaplan–Meier curves to describe the effect of the two treatment methods on the conception rates based on the time from randomization to conception. And we depicted the Kaplan–Meier curves according to age, BMI, HOMA-IR, and FAI stratification.

All data were analyzed using R (http://www.r-project.org) and Empower Stats 2.2 (X&Y Solutions), and a p value < 0.05 was deemed statistically significant.

### Adverse Events

Serious adverse events were defined as immediately life-threatening events, permanent disability, necessitated or extended inpatient hospitalization, intentional or accidental overdoses.

## Results

### Characteristics of the Patients

**Figure 1:**
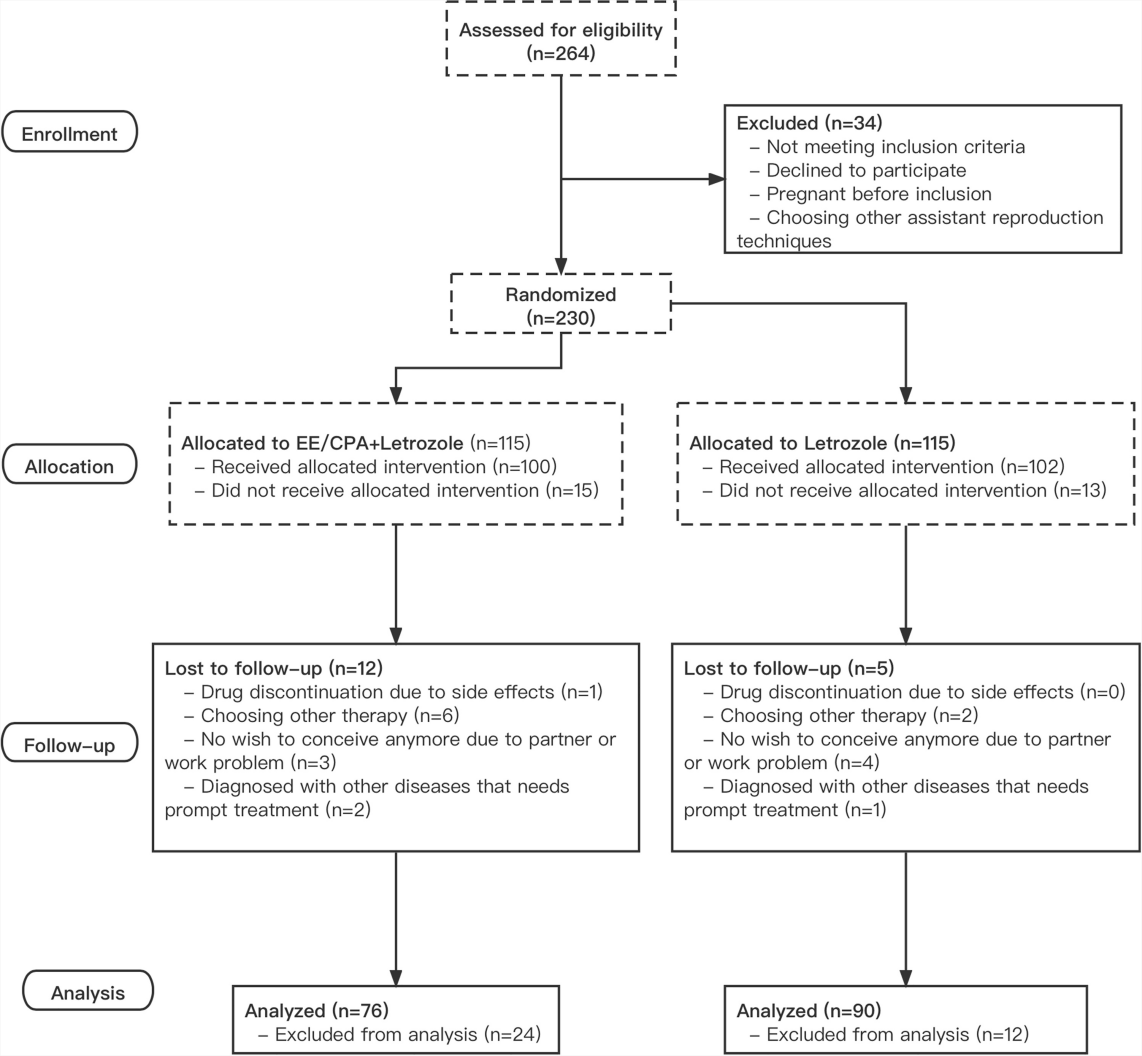
Study flow of the study.

A total of 230 patients with PCOS were randomly allocated to one of the two treatment groups, with 115 patients each in the letrozole and EE/CPA+ letrozole groups. The last patient recruited in the trial completed the last ovulation cycle on December 20, 2020, and the final delivery occurred on September 20, 2021. A total of 39 of 115 patients in the EE/CPA+ letrozole group (33.9%) and 29 of 115 in the letrozole group (21.7%) dropped out or removed from further analysis. As a result, a total of 76 patients in the EE/CPA+ letrozole group and 90 patients in the letrozole group were included in the final analysis (**Figure 1**).

The clinical and demographic characteristics of the patients are listed in **Table 1**, and the two groups were well matched at baseline. Approximately half of the recruited patients in the two groups had a bachelor’s degree, and 40% of them had an income of > 5000RMB per month. Overall, the two groups had similar results in glucose, lipid, thyroid hormone, and sex hormone profiles at baseline (fasting glucose, fasting insulin, total cholesterol, LDL-C, T3, TSH, FSH, LH, total testosterone, SHBG, FAI, and AMH, P>0.05).

**Table 1:** Baseline Characteristics of participants.

Group	letrozole	EE/CPA+letrozole	P-value
**N**	90	76	
**Demographic and anthropometric data**			
Age (y)	28.08 ± 3.19	28.76 ± 3.32	0.233
BMI	2.92 ± 2.77	22.92 ± 2.54	0.774
WHR	0.85 ± 0.08	0.83 ± 0.09	0.224
Nationality			0.484
Han nationality	80 (88.89%)	70 (92.11%)	
Minority nationality	10 (11.11%)	6 (7.89%)	
Education			0.069
High school and below	31 (34.44%)	14 (18.42%)	
Bachelor’s degree	45 (50.00%)	47 (61.84%)	
Post-Graduate and above	14 (15.56%)	15 (19.74%)	
Occupation			0.104
Office staff	49 (54.44%)	54 (71.05%)	
Teachers/lawyers/medical staff	11 (12.22%)	5 (6.58%)	
Workers	12 (13.33%)	4 (5.26%)	
Freelance	18 (20.00%)	13 (17.11%)	
Monthly income (RMB)			0.418
<3000	29 (32.22%)	21 (27.64%)	
3000-5000	25 (27.78%)	22 (28.95%)	
5000-10000	23 (25.56%)	15 (19.74%)	
>10000	13 (14.44%)	18 (23.68%)	
Smoke	4 (4.60%)	4 (5.41%)	0.814
Parity	0.13 ± 0.37	0.08 ± 0.27	0.369
Gravidity	0.40 ± 0.71	0.49 ± 0.95	0.895
Age of menarche (y)	14.40 ± 3.42	13.40 ± 1.42	0.107
Longest length of menstrual cycle (days)	78.95 ± 48.95	80.69 ± 70.70	0.329
Hormonotherapy history	1 (1.11%)	1 (1.32%)	0.904
Endometrial thickness (cm)	0.72 ± 0.87	1.14 ± 1.73	0.062
**Clinical assessments of PCOS**			
m-FS	5.25 ± 4.31	5.05 ± 4.33	0.661
Alopecia	30 (33.33%)	14 (18.42%)	0.052
Acanthosis nigricans	6 (6.67%)	4 (5.26%)	0.705
Acne			0.669
mild	59 (65.56%)	52 (68.42%)	
moderate	10 (11.11%)	6 (7.89%)	
moderate to severe	16 (17.78%)	16 (21.05%)	
severe	5 (5.56%)	2 (2.63%)	
**Endocrine and biochemical measurements**			
T (ng/ml)	0.70 ± 0.34	0.70 ± 0.32	0.848
SHBG (nmol/L)	40.22 ± 27.74	51.79 ± 44.42	0.088
FAI	8.71 ± 7.36	8.88 ± 11.31	0.919
DHEA.S (ng/mL)	2595.09 ± 1403.48	2627.53 ± 1187.01	0.785
LH (IU/L)	11.60 ± 6.32	13.86 ± 9.67	0.145
FSH (IU/L)	6.81 ± 2.18	6.81 ± 2.13	0.848
LH/FSH	1.85 ± 1.05	2.03 ± 1.12	0.218
AMH (ng/mL)	10.06 ± 4.95	8.94 ± 5.22	0.945
TC (mmol/L)	4.77 ± 0.92	4.73 ± 0.78	0.437
LDL.C (mmol/L)	2.98 ± 0.79	2.90 ± 0.70	0.664
T3 (pg/mL)	3.79 ± 1.04	3.83 ± 1.02	0.768
TSH (μIU/mL)	8.35 ± 53.53	2.20 ± 1.15	0.169
Fasting blood-glucose (mmol/L)	5.54 ± 4.52	4.95 ± 0.75	0.541
OGTT 2-hour glucose (mmol/L)	6.20 ± 1.22	6.93 ± 6.38	0.822
Fasting insulin (μIU/mL)	12.24 ± 8.99	10.31 ± 5.16	0.340
OGTT 2-hour insulin (μIU/mL)	70.96 ± 59.19	64.55 ± 55.73	0.223
HOMA.IR	2.90 ± 2.91	2.34 ± 1.39	0.308

EE/CPA, ethinylestradiol/cyproterone acetate; BMI, body mass index; WHR, waist-to-hip ratio; RMB, ren min bi; m-FS, modified Ferriman–Gallwey; T, testosterone; SHBG, sex hormone binding globulin; FAI, free androgen index; DHEA.S, sulfated dehydroepiandrosterone; LH, luteinizing hormone; FSH, follicle-stimulating hormone; AMH, anti-Mullerian hormone; TC, total cholesterol; LDL.C, low-density lipoprotein cholesterol; T3, triiodothyronine; TSH, thyroid stimulating hormone; OGTT, 75-g oral glucose tolerance test; HOMA.IR, homeostasis model assessment for insulin resistance.

### Primary and Secondary Outcomes

As shown in **Table 2**, there were no significant differences in the conception (44/90 [48.89%] *vs*. 42/76 [55.26%], RR= 1.29 [0.70,2.38], P=0.713), ongoing pregnancy (33/90 [36.67%] *vs*. 33/76 [43.42%], RR=1.33 [0.71,2.47], P=0.376), and live birth rates (32/90 [35.56%] *vs*. 31/76 [40.79%], RR=1.25 [0.67, 2.34], P=0.489) between the letrozole and EE/CPA+ letrozole groups. Additionally, no statistical difference in the cumulative ovulation rate was observed between the letrozole and EE/CPA+ letrozole groups (206/254 [81.10%] *vs*. 169/205 [82.44%], RR=1.09 [0.68,1.76], P=0.713).

**Table 2:** Primary outcomes and secondary outcomes of patients of EE/CPA + letrozole and letrozole groups.

Group	letrozole	EE/CPA + letrozole	RR(95%CI)	P-value
**Primary outcomes**				
Ovulation	206/254 (81.10%)	169/205 (82.44%)	1.09 (0.68,1.76)	0.713
Conception	44/90 (48.89%)	42/76 (55.26%)	1.29 (0.70,2.38)	0.413
Ongoing pregnancy	33/90 (36.67%)	33/76 (43.42%)	1.33 (0.71,2.47)	0.376
Live birth	32/90 (35.56%)	31/76 (40.79%)	1.25 (0.67, 2.34)	0.489
**Secondary outcomes**				
Ovulation per treatment cycle				
Cycle 1	66/90 (73.33%)	51/76 (67.11%)	0.74 (0.38,1.45)	0.381
Cycle 2	61/74 (82.43%)	55/65 (84.62%)	1.17 (0.48, 2.89)	0.730
Cycle 3	55/63 (87.30%)	46/46 (100.00%)	Inf. (0.00, Inf)	0.994
Cycle 4	24/27 (88.89%)	17/18 (94.44%)	2.04 (0.20, 21.30)	0.551
Conception per treatment cycle				
Cycle 1	16/90 (17.78%)	11/76 (14.47%)	0.78 (0.34,1.81)	0.566
Cycle 2	11/74 (14.86%)	19/89 (29.23%)	2.37 (1.03,5.45)	**0.043** ^a^
Cycle 3	10/63 (15.87%)	8/46 (17.39%)	1.12 (0.40,3.09)	0.833
Cycle 4	7/27 (25.93%)	4/18 (22.22%)	0.86 (0.21,3.48)	0.830
Letrozole dosage for ovulation (g)	3.03 ± 1.03	3.22 ± 1.34	–	0.301
Letrozole dosage for pregnancy (g)	3.43 ± 1.73	3.69 ± 1.58	–	0.471
Average cycles taken to ovulation (cycles)	1.27 ± 0.52	1.38 ± 0.61	–	0.215
Average cycles taken to pregnancy (cycles)	2.14 ± 1.08	2.10 ± 0.93	–	0.840
Average time taken to pregnancy (days)	99.98 ± 67.31	178.60 ± 55.04	–	**<0.001** ^b^

EE/CPA, ethinylestradiol/cyproterone acetate; RR, risk ratio; Inf, infinite. ^a^The EE/CPA+ letrozole group had a higher pregnancy rate in the second treatment cycle than the letrozole group (Cycle 2: 19/ 89 [29.23%] vs. 11/74 [14.86%], RR=2.37 [1.03,5.45], P=0.0431). ^b^The average time taken to pregnancy in the EE/CPA+ letrozole group was significantly longer than that in the letrozole group (178.60 ± 55.04 vs. 99.98 ± 67.31, P< 0.001).

The EE/CPA+ letrozole group had a higher pregnancy rate in the second treatment cycle than the letrozole group (Cycle 2: 19/89 [29.23%] *vs*. 11/74 [14.86%], RR=2.37 [1.03,5.45], **P**=0.0431).However, the letrozole and EE/CPA+ letrozole groups showed no statistical differences in the secondary outcomes, including ovulation rate per treatment cycle (Cycle 1: 66/90 [73.33%] *vs*. 51/76[67.11%], **P**=0.381; Cycle 2: 61/74 [82.43%] *vs*. 55/65[84.62%], **P**=0.730; Cycle 3: 55/63 [87.30%] *vs*. 46/46 [100.00%], **P**=0.994; Cycle 4: 24/27 [88.89%] *vs*. 17/18 [94.44%], **P**=0.551), conception rate per treatment cycle (Cycle 1: 16/90 [17.78%] *vs*. 11/76 [14.47%], **P**=0.566; Cycle 3: 10/63 [15.87%] *vs*. 8/46 [17.39%], **P**=0.833; Cycle 4: 7/27 [25.93%] *vs*. 4/18 [22.22%], **P**=0.830), average cycles taken to ovulation (1.38 ± 0.61 *vs*. 1.27 ± 0.52, **P**=0.215), letrozole dosage for ovulation (3.03 ± 1.03 *vs*. 3.22 ± 1.34, **P**=0.301), average cycles taken to pregnancy (2.14 ± 1.08 *vs*. 2.10 ± 0.93, **P**=0.84), and letrozole dosage for pregnancy (3.43 ± 1.73 *vs*. 3.69 ± 1.58, **P**=0.471). To note, the average time taken to pregnancy in the EE/CPA+ letrozole group was significantly longer than that in the letrozole group (178.60 ± 55.04 *vs*. 99.98 ± 67.31, **P**< 0.001), as a result of extended 3 months of antiandrogen pretreatment in the EE/CPA+ letrozole group.

We also analyzed the effect of COC pretreatment based on age, BMI, HOMA-IR, FAI stratification. No significant differences in ovulation rate per cycle, cumulative ovulation, conception, ongoing pregnancy, and live birth rate were observed between the two treatment groups according to age (**Table 3**), BMI (**Table 4**), HOMA-IR (**Table 5**), and FAI stratification (**Table 6**).

**Table 3:** Primary outcomes and secondary outcomes of patients of EE/CPA + letrozole and letrozole groups based on age stratification.

Age (y)	<30		≥30	
N(%)	102 (61.4%)		64 (38.6%)	
**Primary outcomes**				
Ovulation	1.02 (0.56,1.85)	0.946	1.10 (0.49, 2.49)	0.812
Conception	1.48 (0.67,3.29)	0.332	1.13 (0.42, 3.04)	0.802
Ongoing pregnancy	1.55 (0.70, 3.42)	0.275	1.16 (0.40, 3.40)	0.785
Live birth	1.39 (0.63, 3.07)	0.416	1.16 (0.40, 3.40)	0.785
**Secondary outcomes**				
Ovulation per treatment cycle				
Cycle 1	0.73 (0.31, 1.67)	0.451	0.72 (0.23, 2.23)	0.565
Cycle 2	1.38 (0.45, 4.26)	0.574	0.75 (0.15, 3.69)	0.723
Cycle 3	Inf. (0.00, Inf)	0.994	inf. (0.00, Inf)	0.996
Cycle 4	0.33 (0.02, 6.19)	0.461	inf. (0.00, Inf)	0.997
Conception per treatment cycle				
Cycle 1	0.89 (0.34, 2.32)	0.812	0.64 (0.10, 4.14)	0.644
Cycle 2	2.98 (0.97, 9.10)	0.056	1.75 (0.50, 6.14)	0.386
Cycle 3	2.27 (0.61, 8.46)	0.223	0.38 (0.07, 2.20)	0.280
Cycle 4	0.36 (0.04, 3.70)	0.391	3.00 (0.25, 35.33)	0.383

EE/CPA, ethinylestradiol/cyproterone acetate; Inf, infinite.

**Table 4:** Primary outcomes and secondary outcomes of patients of EE/CPA + letrozole and letrozole groups based on BMI stratification.

BMI (kg/m^2^)	18.5–23.9		24–28	
N (%)	112 (67.5%)		54 (32.5%)	
**Primary outcomes**				
Ovulation	0.97 (0.52, 1.78)	0.910	1.38 (0.64, 2.99)	0.411
Conception	1.24 (0.58, 2.63)	0.583	1.54 (0.52, 4.60)	0.436
Ongoing pregnancy	1.02 (0.48, 2.16)	0.962	2.69 (0.82, 8.85)	0.104
Live birth	1.09 (0.51, 2.31)	0.826	1.94 (0.58, 6.52)	0.2832
**Secondary outcomes**				
Ovulation per treatment cycle				
Cycle 1	0.68 (0.30, 1.56)	0.360	0.89 (0.29, 2.78)	0.847
Cycle 2	1.00 (0.31, 3.25)	1.000	1.50 (0.36, 6.18)	0.575
Cycle 3	inf. (0.00, Inf)	0.996	inf. (0.00, Inf)	0.994
Cycle 4	inf. (0.00, Inf)	0.995	0.00 (0.00, Inf)	0.998
Conception per treatment cycle				
Cycle 1	0.72 (0.27, 1.90)	0.504	1.09 (0.20, 5.94)	0.923
Cycle 2	2.47 (0.95, 6.45)	0.065	2.42 (0.40, 14.69)	0.336
Cycle 3	0.99 (0.25, 3.88)	0.983	1.27 (0.27, 5.92)	0.764
Cycle 4	0.80 (0.16, 3.94)	0.784	1.25 (0.06, 26.87)	0.8866

EE/CPA, ethinylestradiol/cyproterone acetate; BMI, body mass index; Inf, infinite.

**Table 5:** Primary outcomes and secondary outcomes of patients of EE/CPA + letrozole and letrozole groups based on HOMA.IR stratification.

HOMA.IR	<2.63		≥2.63	
N (%)	93 (56.0%)		55 (44.0%)	
**Primary outcomes**				
Ovulation	0.91 (0.47, 1.77)	0.776	1.68 (0.78, 3.63)	0.187
Conception	1.26 (0.56, 2.86)	0.582	1.21 (0.42, 3.51)	0.729
Ongoing pregnancy	1.22 (0.54, 2.79)	0.635	1.31 (0.42, 4.06)	0.637
Live birth	1.02 (0.44, 2.34)	0.967	1.55 (0.49, 4.88)	0.457
**Secondary outcomes**				
Ovulation per treatment cycle				
Cycle 1	0.66 (0.26, 1.66)	0.375	1.23 (0.40, 3.78)	0.717
Cycle 2	0.61 (0.16, 2.36)	0.475	1.85 (0.46, 7.48)	0.387
Cycle 3	inf. (0.00, Inf)	0.996	inf. (0.00, Inf)	0.994
Cycle 4	inf. (0.00, Inf)	0.995	0.00 (0.00, Inf)	0.998
Conception per treatment cycle				
Cycle 1	0.77 (0.24, 2.42)	0.654	0.55 (0.12, 2.45)	0.429
Cycle 2	2.83 (0.95, 8.46)	0.062	2.44 (0.40, 14.91)	0.333
Cycle 3	0.92 (0.25, 3.31)	0.896	1.27 (0.22, 7.20)	0.790
Cycle 4	0.65 (0.10, 4.29)	0.655	2.00 (0.18, 22.06)	0.571

EE/CPA, ethinylestradiol/cyproterone acetate; HOMA-IR, homeostasis model assessment for insulin resistance; Inf, infinite.

**Table 6:** Primary outcomes and secondary outcomes of patients of EE/CPA + letrozole and letrozole groups based on FAI stratification.

FAI		Low (0.54-2.57)		Middle (2.72-6.35)		High (6.37-71.57)	
N		47		47		47	
**Primary outcomes**							
Ovulation	1.21 (0.49, 2.99)		0.678	1.53 (0.55, 4.29)	0.416	0.47 (0.16,1.36)	0.162
Conception	0.78 (0.24, 2.59)		0.689	0.97 (0.30, 3.15)	0.959	2.14 (0.63, 7.27)	0.221
Ongoing pregnancy	0.48 (0.15, 1.56)		0.223	1.28 (0.37, 4.40)	0.696	2.37 (0.71, 7.92)	0.159
Live birth	0.41 (0.12, 1.34)		0.141	1.02 (0.29, 3.60)	0.978	2.78 (0.82, 9.39)	0.100
**Secondary outcomes**							
Ovulation per treatment cycle							
Cycle 1	0.59 (0.15, 2.34)		0.457	0.35 (0.10, 1.24)	0.103	1.84 (0.52, 6.53)	0.348
Cycle 2	1.58 (0.33, 7.56)		0.565	0.76 (0.10, 6.01)	0.796	1.94 (0.33, 11.56)	0.465
Cycle 3	inf. (0.00, Inf)		0.997	inf. (0.00, Inf)	0.997	inf. (0.00, Inf)	0.995
Cycle 4	1.00 (0.00, Inf)		1.000	inf. (0.00, Inf)	0.997	–	–
Conception per treatment cycle							
Cycle 1	0.70 (0.15, 3.20)		0.641	0.64 (0.10, 3.89)	0.627	0.48 (0.09, 2.68)	0.403
Cycle 2	1.60 (0.39, 6.62)		0.517	4.04 (0.68, 23.94)	0.124	3.50 (0.56, 22.03)	0.182
Cycle 3	0.46 (0.06, 3.35)		0.444	0.79 (0.06, 9.71)	0.855	2.13 (0.42, 10.75)	0.362
Cycle 4	0.80 (0.08, 8.47)		0.853	0.00 (0.00, Inf)	0.996	–	–

EE/CPA, ethinylestradiol/cyproterone acetate; FAI, free androgen index; Inf, infinite.

**Figure 2:**
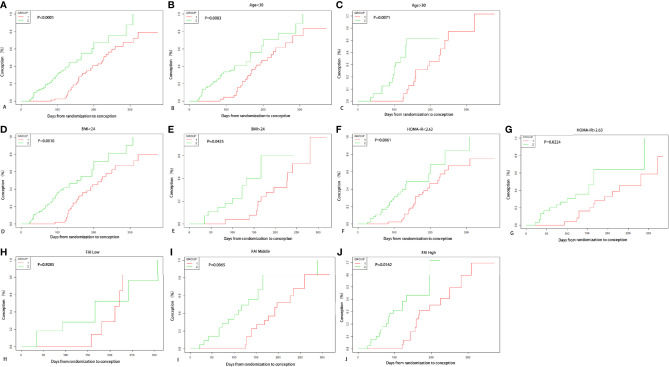
Kaplan-Meier Curves for conception. Conception rate varying with days from randomization to outcome of two treatment groups are shown in **(A)**, and conception rate of two groups according to age subgrouping in **(B, C)**, and according to BMI subgrouping in **(D, E)**, and according to HOMA-IR subgrouping in **(F, G)**, and according to FAI tertile subgrouping in **(H–J)**.

According to Kaplan-Meier analysis (**Figure 2**), the conception rate of letrozole group was higher than that of COC pretreatment group when considering the longer period from randomization to outcome event in EE/CPA+ letrozole group (**P**<0.0001), which was also observed in age subgroups (age<30: **P**=0.0083, age≥30: **P**=0.0071), BMI subgroups (BMI<24: P=0.0010, BMI≥24: **P**=0.0425), HOMA-IR subgroups (HOMA-IR<2.63: **P**=0.0061, HOMA-IR≥2.63: **P**=0.0224), and FAI subgroups (Middle FAI (2.72-6.35): **P**=0.0065, High FAI (6.37-71.57): **P**=0.0162).

### Adverse Events

None of the patients in our study experienced serious adverse events. The adverse events reported in our study were transient and mild. During COC pretreatment in the EE/CPA+ letrozole group, one patient complained of diarrhea, two had headache, and eight had irregular vaginal bleeding. In addition, during the period of ovulation induction, there were two cases of nausea, one case of headache, and four cases of breast distending pain in the letrozole group. While in the EE/CPA+ letrozole group, three patients reported nausea, and two had breast distending pain. There were no significant differences in adverse events between the letrozole and EE/CPA+ letrozole groups (7/90 [7.7%] and 5/76 [6.5%], respectively, **P**>0.05).

There was one patient in EE/CPA+ letrozole group undergoing induced abortion in the second trimester due to twin-twin transfusion syndrome (TTTS). And multiple pregnancy rates in the letrozole and EE/CPA+ letrozole groups were 1.3% (1/76) and 1.1% (1/90), respectively. Gestational diabetes and pre-eclampsia were shown to be prevalent in 2.6% (2/76) and 3.33% (3/90) of the live deliveries in the two groups, respectively.

## Discussion

In this randomized clinical trial, we investigated the effectiveness of COC antiandrogenic pretreatment prior to ovulation induction in PCOS patients with hyperandrogenism by comparing the pregnancy outcomes of the patients receiving COC pretreatment before ovulation induction and those immediately receiving letrozole treatment. The results showed no differences in cumulative ovulation, conception, ongoing pregnancy, and live birth rates between the two groups, indicating the equal effectiveness of both treatment strategies. Given the time and psychological costs of delaying treatment, prompt ovulation induction can be advised for those patients with conception plans.

As excessive androgen levels cause a series of harmful reproduction-related complications in women with PCOS, antiandrogenic therapy may also have the potential to be an auxiliary method in enhancing pregnancy outcomes. COCs are common therapeutic agents for reducing androgen expression in patients with PCOS who have no pregnancy intentions (30–32). However, their effect on improving pregnancy outcomes in patients with PCOS with anovulatory dysfunction remains uncertain. The COC pretreatment is also inhibited by the dilemma of initiate prompt ovulation induction or improving the endocrine environment prior to infertility therapy for patients with PCOS. On one hand, EE/CPA pretreatment can reduce androgen levels and improve endocrine and metabolic status in patients with PCOS, with potential benefits for pregnancy outcomes (33). On the other hand, postponing treatment may cause impatience and anxiety in patients, which might result in an adverse effect. As a result, the rationale for providing COC pretreatment and delaying immediate infertility therapy in clinical practice is in great demand for clinicians and patients. Yet, the effect of COC pretreatment prior to ovulation induction has seldom been discussed.

One single-centered prospective study investigated the role of COC pretreatment in patients with PCOS with clomiphene resistance, of which the findings could not be generalized to other populations (34). The first adequately controlled study to explore COC pretreatment before inducing ovulation in patients with PCOS was performed by Paloma et al. (9). In their study, the experimental group received one cycle of COC pretreatment, comprising of 20 mg of ethinyl estradiol (E2) and 75 mg of gestodene. All patients underwent controlled ovarian stimulation using a low-dose stet-up highly purified urine FSH (u-FSH) protocol. No significant improvement in pregnancy outcomes, including pregnancy and live birth rates, was identified in patients receiving COC pretreatment. Later in 2016, Lergo et al. combined two randomized concurrently conducted clinical trials and discovered no difference in ovulation and live birth rates between COC pretreatment and immediate clomiphene therapy. In this *post hoc* study, the sample size for the COC group was relatively small, with 47 patients enrolled (35). Our findings that the experimental and control groups had virtually equal ovulation and pregnancy rates are consistent with those earlier studies, indicating that androgen suppression by EE/CPA pretreatment offers minimal reproductive advantage for PCOS patients with hyperandrogenism.

Kaplan-Meier analysis of our study have shown that the conception rate of letrozole group was higher than that of COC pretreatment group. However, it is challenging to evaluate benefits of two groups, due to the life table analyses employed in the primary outcome (ovulation induction in EE/CPA+ letrozole group started later than that in the letrozole group) (36). Furthermore, because of the greater conception rate of immediate therapy compared to that during the intervention of the COC pretreatment, the Kaplan-Meier curve would likely be biased in favor of immediate treatment (letrozole group).

There are various possible explanations for the result that antiandrogenic pretreatment with EE/CPA presented no additional fertility benefits. Firstly, the progestin administration with COC pretreatment might be correlated with decreased conception rates, as reported in a secondary analysis of PCOSI data by Legro and colleagues (37). Progestin, on the one hand, has adverse effects on the endometrium directly, which may be detrimental to subsequent conception. On the other hand, progestin exposure may affect the hypothalamic-pituitary-ovarian axis that modifies the sex hormones in women, thus impacting later conception indirectly (38). Another possibility is that the benefit of androgen reduction on pregnancy outcomes is insufficient to compensate for the potential unfavorable effect caused by the “lost” time of three consecutive cycles of EE/CPA antiandrogenic therapy.

The prospective and multi-centered design constituted the main strength of the study. Before initiating recruitment, the investigators from all participating centers received standard training programs about the study protocol and blinding methods. A representative from the corresponding center supervises each center’s research processes and progress to guarantee that the study was conducted in strict compliance with the protocol enacted by experts. Compared with previous studies, the randomly controlled and multi-centered design of the study reinforced the conclusion and could serve as a reference in clinical practice.

This study has several limitations. First, we did not re-assess the androgen levels of the individuals after EE/CPA pretreatment for 3 months, considering the excellent antiandrogenic effect of EE/CPA in patients with PCOS and cost reduction in this study (39). Additionally, patients of the two groups received therapies with different time durations and fertility guidance. For those who had direct ovulation induction, routine intercourse was instructed in the cycles, which was not required for those who had COC pretreatment in the first 3 months. As a result, this study used an open-label design, in which patients and research assistants could not be blinded to the treatment groups. However, the doctors who were in charge of recruiting patients as well as the data analysts were blinded to the random allocation and specific grouping. Moreover, the high dropout rate constitutes another notable shortcoming of this research, especially in the COC pretreatment group. The dropout rate in our study was similar to that of other studies involving ovulation induction (40). We speculated that the high dropout rate could be related to the discouragement and anxiety of couples who have undergone long periods of preparation for pregnancy. The couples tended to resort to other assistant reproduction techniques after multiple failures. The strict anti-epidemic quarantine policy in China also contributed to treatment discontinuation and changes in pregnancy plans.

## Conclusion

Our study showed that COC pretreatment was not superior to direct letrozole-induced ovulation therapy in improving ovulation and pregnancy outcomes in women with PCOS. Based on the results of this multicentered randomized study, there is no benefit to antiandrogenic pretreatment before ovulation induction in PCOS patients with hyperandrogenism. With the design and implementation process of this study, we believe that the results are representative and can be a reference for clinical practice.

## Data Availability Statement

The datasets presented in this study can be found in online repositories. The names of the repository/repositories and accession number(s) can be found below: Resman platform (http://www.medresman.org.cn).

## Ethics Statement

The studies involving human participants were reviewed and approved by The Institutional Review Board (IRB) of Peking Union Medical College Hospital. The patients/participants provided their written informed consent to participate in this study.

## Author Contributions

ZC, JT, HW, BZ, JL, and GH collected and validated the patient data. ZC analyzed and interpreted the patient data. ZS managed the data and edited the manuscript and QY supervised the study and revised the manuscript. All authors contributed to the article and approved the submitted version.

## Funding

Supported by the National Key Research and Development Program (2018YFC1002105) and the Non-profit Central Research Institute Fund of Chinese Academy of Medical Sciences (NO. 2020-PT320-003).

## Conflict of Interest

The authors declare that the research was conducted in the absence of any commercial or financial relationships that could be construed as a potential conflict of interest.

## Publisher’s Note

All claims expressed in this article are solely those of the authors and do not necessarily represent those of their affiliated organizations, or those of the publisher, the editors and the reviewers. Any product that may be evaluated in this article, or claim that may be made by its manufacturer, is not guaranteed or endorsed by the publisher.
